# Assessment of Automated Identification of Phases in Videos of Cataract Surgery Using Machine Learning and Deep Learning Techniques

**DOI:** 10.1001/jamanetworkopen.2019.1860

**Published:** 2019-04-05

**Authors:** Felix Yu, Gianluca Silva Croso, Tae Soo Kim, Ziang Song, Felix Parker, Gregory D. Hager, Austin Reiter, S. Swaroop Vedula, Haider Ali, Shameema Sikder

**Affiliations:** 1 Department of Computer Science, Johns Hopkins University, Baltimore, Maryland; 2 Malone Center for Engineering in Healthcare, Johns Hopkins University, Baltimore, Maryland; 3 Wilmer Eye Institute, Johns Hopkins University School of Medicine, Baltimore, Maryland

## Abstract

**Importance:**

Competence in cataract surgery is a public health necessity, and videos of cataract surgery are routinely available to educators and trainees but currently are of limited use in training. Machine learning and deep learning techniques can yield tools that efficiently segment videos of cataract surgery into constituent phases for subsequent automated skill assessment and feedback.

**Objective:**

To evaluate machine learning and deep learning algorithms for automated phase classification of manually presegmented phases in videos of cataract surgery.

**Design, Setting, and Participants:**

This was a cross-sectional study using a data set of videos from a convenience sample of 100 cataract procedures performed by faculty and trainee surgeons in an ophthalmology residency program from July 2011 to December 2017. Demographic characteristics for surgeons and patients were not captured. Ten standard labels in the procedure and 14 instruments used during surgery were manually annotated, which served as the ground truth.

**Exposures:**

Five algorithms with different input data: (1) a support vector machine input with cross-sectional instrument label data; (2) a recurrent neural network (RNN) input with a time series of instrument labels; (3) a convolutional neural network (CNN) input with cross-sectional image data; (4) a CNN-RNN input with a time series of images; and (5) a CNN-RNN input with time series of images and instrument labels. Each algorithm was evaluated with 5-fold cross-validation.

**Main Outcomes and Measures:**

Accuracy, area under the receiver operating characteristic curve, sensitivity, specificity, and precision.

**Results:**

Unweighted accuracy for the 5 algorithms ranged between 0.915 and 0.959. Area under the receiver operating characteristic curve for the 5 algorithms ranged between 0.712 and 0.773, with small differences among them. The area under the receiver operating characteristic curve for the image-only CNN-RNN (0.752) was significantly greater than that of the CNN with cross-sectional image data (0.712) (difference, −0.040; 95% CI, −0.049 to −0.033) and the CNN-RNN with images and instrument labels (0.737) (difference, 0.016; 95% CI, 0.014 to 0.018). While specificity was uniformly high for all phases with all 5 algorithms (range, 0.877 to 0.999), sensitivity ranged between 0.005 (95% CI, 0.000 to 0.015) for the support vector machine for wound closure (corneal hydration) and 0.974 (95% CI, 0.957 to 0.991) for the RNN for main incision. Precision ranged between 0.283 and 0.963.

**Conclusions and Relevance:**

Time series modeling of instrument labels and video images using deep learning techniques may yield potentially useful tools for the automated detection of phases in cataract surgery procedures.

## Key Points

**Question:**

Are deep learning techniques sufficiently accurate to classify presegmented phases in videos of cataract surgery for subsequent automated skill assessment and feedback?

**Findings:**

In this cross-sectional study including videos from a convenience sample of 100 cataract procedures, modeling time series of labels of instruments in use appeared to yield greater accuracy in classifying phases of cataract operations than modeling cross-sectional data on instrument labels, spatial video image features, spatiotemporal video image features, or spatiotemporal video image features with appended instrument labels.

**Meaning:**

Time series models of instruments in use may serve to automate the identification of phases in cataract surgery, helping to develop efficient and effective surgical skill training tools in ophthalmology.

## Teaser

This cross-sectional study evaluates machine learning and deep learning algorithms for automated phase classification of manually presegmented videos of cataract procedures.

## Introduction

Learning to competently perform cataract surgery is a universal and essential aspect of graduate surgical training in ophthalmology. In 2010, an estimated 2.8 million operations were performed in the United States to replace the opaque natural lens with a clear artificial lens, which is the cornerstone of treatment for cataracts.^1^ In Medicare patients with cataracts, cataract surgery is associated with a reduction in risk of all-cause mortality by 27% (95% CI, 26%-28%).^2^ While approximately 1 in 6 US adults older than 40 years are estimated to have cataracts,^3,4^ its prevalence is estimated to rise to 30.1 million by 2020^3^ and nearly double to approximately 50 million by 2050.^4^ To meet this demand, ophthalmology residency programs must efficiently train surgeons to competently perform cataract operations. However, a 2006 survey of ophthalmologists who had been in practice for 5 years or less showed that 14% of respondents felt unprepared to perform the procedure.^5^ In another survey of residency program directors in 2006, an average of 1 in 10 residents had difficulty learning cataract surgery and required remedial educational measures.^6^ Furthermore, the learning curve for cataract surgery is associated with an increased incidence of intraoperative adverse events for residents.^7^ Clearly, to adequately train the next generation of ophthalmologists, surgical educators must develop curricula that include systematic assessments of skill and competency to delegate commensurate responsibility to preserve patient safety.

Despite recent paradigm shifts in graduate surgical training, the effectiveness of residency training in ophthalmology is limited by subjective and inefficient tools for assessing intraoperative technical skill and competency. Graduate education in ophthalmology has evolved toward competency-based training following a mandate from the Accreditation Council for Graduate Medical Education. Accordingly, the Accreditation Council for Graduate Medical Education and the American Board of Ophthalmology developed milestones for different procedures, including cataract surgery, to evaluate trainees’ competency.^8^ However, this framework does not specify how to quantitatively assess residents’ intraoperative technical skill and competency. Consequently, residency training curricula continue to rely on either unstandardized and unreliable measures or structured rating scales to assess intraoperative technical skill. Several structured rating scales have been developed to assess technical skill for cataract surgery, but all of them provide the observing rater’s subjective opinion.^9^ Furthermore, these structured rating scales require considerable time from faculty surgeons to be routinely incorporated within training curricula. Thus, lack of access to objective and valid measures that can be efficiently implemented to evaluate residents’ performance limits the effectiveness of training curricula.

In cataract surgery, technical skill is typically assessed within the procedure’s constituent tasks or phases (eg, corneal incision, capsulorrhexis, phacoemulsification).^9^ Technologies that segment, classify, and analyze readily available microscopic videos of cataract operations can enable data-driven, objective, and valid assessments and feedback. This is now possible owing to advances in computer vision, machine learning, and deep learning. Broadly, 2 approaches are available to obtain videos of the phases of cataract surgical procedures. The first, content-based video retrieval, involves matching a video to other, similar videos in a data set.^10,11,12,13^ In this approach, videos are first transformed into fixed-dimensional feature representations using computer vision techniques and then evaluated with distance metrics within the feature space.^10,11,12,13^ The second approach consists of decomposing a procedure video into its constituent phases (segmentation)^14^ and assigning each segment a phase label (classification).^15,16^ Methods for the second approach include computer vision techniques^14^ and deep learning.^14,16^ Our objective in this study was to evaluate different machine learning and deep learning algorithms to classify a given video segment, ie, to identify the phase within manually presegmented videos, of cataract surgery.

## Methods

### Data and Reference Standard

Our data set included videos of 100 cataract surgery procedures (29 performed by a faculty surgeon and 71 by a trainee surgeon under supervision) that took place between July 2011 and December 2017, captured at the Wilmer Eye Institute, Johns Hopkins University, Baltimore, Maryland. This study was approved by the Johns Hopkins University School of Medicine Institutional Review Board. Informed consent was not required for this retrospective cohort of deidentified videos captured for training purposes. This study followed the Strengthening the Reporting of Observational Studies in Epidemiology (STROBE) reporting guideline.

We studied 10 phases in cataract surgery: (1) side incision, (2) main incision, (3) capsulorrhexis, (4) hydrodissection, (5) phacoemulsification, (6) cortical removal, (7) lens insertion, (8) ophthalmic viscosurgical device removal, (9) wound closure (corneal hydration), and (10) wound closure (suturing incision). Using prespecified definitions for the 10 phases, 1 physician (S.S.V.) annotated the start and end of the phases in each video. We counted unsuccessfully performed phases and phases repeated in part within other phases, such as a failed lens insertion or the rotation of the lens with a hook during wound closure, as a distinct instance of that phase. The manual annotations served as the reference standard to train and test our algorithms.

Within each phase, another trained annotator (Z.S. or S.S.V.) marked the beginning and end of the use of the following instruments: paracentesis blade, forceps, crescent blade, keratome blade, cystotome, Utrata forceps, hydrodissection cannula, phacoemulsification probe, irrigation-aspiration handpiece, intraocular lens injector, Sinskey hook, chopper, suture, and needle driver. The annotations were a time series of instrument labels, ie, video images were labeled with either the name(s) of instrument(s) in use or a label for no instrument. To generate a cross-section of labels for each phase instance, we sampled frames with a unique combination of instruments used during the phase. This yielded a 14-dimensional binary vector for each phase instance with the instrument(s) used coded as 1 and the rest as 0.

### Algorithms

**Figure 1.**
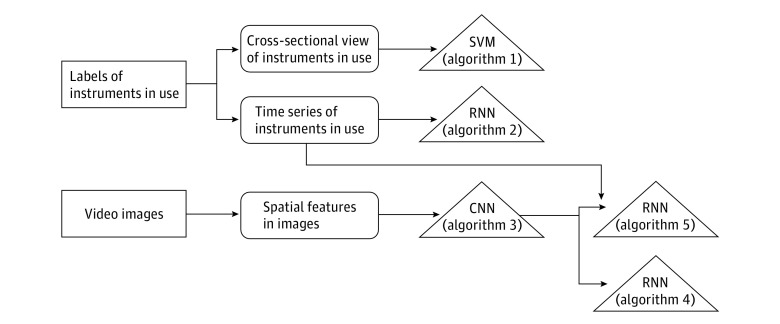
Algorithms Evaluated for Classification of Phases in Cataract Surgery CNN indicates convolutional neural network; RNN, recurrent neural network; SVM, support vector machine.

Figure 1 illustrates the data sources, features, and 5 algorithms evaluated in this study. Full technical implementation details of the algorithms are available in the eAppendix in the Supplement.

The first algorithm we evaluated was a support vector machine (SVM) using the cross-sectional binary feature of instrument labels. We used a linear kernel SVM as a 1-vs-all multiclass classifier and trained 1 SVM for each of the 10 phases (vs all other phases). For a given test instance, we used all 10 SVMs to predict a phase label. We identified the phase-specific SVM in which the test instance showed the greatest separation from the hyperplane and assigned the corresponding phase label as the prediction.

The second algorithm we evaluated was a recurrent neural network (RNN) using instrument labels as time series data. We used a simple RNN architecture with multiple long short-term memory (LSTM) nodes. The LSTM allowed us to control how much information from the previous time steps was retained at each node and thus to model temporal relations across a window of time. We used a single LSTM layer along with a fully connected layer that outputs a feature vector of length 128, which is a temporal encoding of the video for the phase. To this encoding, we applied a softmax prediction layer of 10 dimensions (corresponding to the 10 phase labels). The phase label with the highest softmax probability was the predicted label.

The third algorithm we evaluated was a convolutional neural network (CNN) using spatial features within images. We used an existing CNN architecture called SqueezeNet (DeepScale; University of California, Berkeley; and Stanford University) to model spatial features across images within a phase and discriminate across phases.^17^ Convolutional neural networks learn local patterns in the data (in this case, context within the surgical field) through convolutions, which involve data transformations within patches of input features to encode specific aspects of the data. SqueezeNet consists of multiple fire modules, each of which contains a series of 1 × 1 convolutional filters (fire2) that squeeze the information, followed by a set of 1 × 1 and 3 × 3 convolutional filters (fire9) that expand it. We trained the CNN with cross-entropy loss for 100 epochs in batches of 128 images and set the initial learning rate to 0.001, decay to 0.0004, and momentum to 0.9. In the third analysis, we extracted a 512-dimensional encoding from the last convolutional layer for each image frame in a video. To this encoding, we applied a softmax prediction layer of 10 dimensions to obtain the predicted label.

The fourth algorithm we evaluated was a CNN-RNN pipeline to model spatiotemporal features across images. To learn a temporal encoding for the entire phase, we concatenated the 512-dimensional encoding obtained from the third algorithm for each image with the corresponding instrument label annotation. We used the concatenated feature to train an LSTM network to learn spatiotemporal patterns that discriminate across phases. The LSTM constituted 512 hidden units to emit a 512-dimensional, temporally average pooled vector as the encoding for the phase. We applied a softmax prediction layer of 10 dimensions to this encoding to obtain the predicted label.

The fifth algorithm we evaluated was a CNN-RNN pipeline to model spatiotemporal features across images with instrument labels added as time-series data. We used the same CNN-RNN pipeline as in the fourth algorithm, but we concatenated instrument labels to the 512-dimensional encoding from the CNN before temporal modeling with an LSTM.

### Experiment Design

We analyzed each algorithm in a separate experiment using a 5-fold cross-validation setup. We randomly assigned procedures to the 5 folds, stratified by the appointment status of the operating surgeon (faculty or trainee), such that differences in the sum of durations between folds was minimized. We iteratively used data from 3 folds to train the algorithm, 1 fold to validate (ie, optimize model parameters, eg, regularization parameters for the SVM), and 1 fold to test it.

### Statistical Analysis

We measured algorithm performance globally across phases using accuracy and area under the receiver operating characteristic curve (AUC) (eTable 1 in the Supplement). For accuracy, we computed the unweighted mean as well as means weighted by frequency of phases and the inverse of variance of phase-specific accuracies. To evaluate phase-level performance, we computed phase-level sensitivity, specificity, and precision. We computed the AUC for a multinomial phase classification task using previously described methods.^18^ For the SVM and CNN (first and third algorithms), the algorithm predicted a phase label for each frame in the video. We used the mode of these frame-level predictions as the phase-level–predicted label to compute accuracy, sensitivity, specificity, and precision and frame-level–predicted probabilities to compute AUC.

Finally, to account for multiple instances of a phase in some videos in our data set, we used multiple outputation to estimate phase-specific metrics and their 95% CIs.^19^ In each outputation, we randomly sampled 1 instance of each phase observed in a given video. To compare algorithm performance, we computed pairwise differences in AUC for each outputation and, based on the asymptotic normality of the multiple outputation estimates, used a normal distribution to obtain the 2-sided *P* value. We applied a Bonferroni correction to consider *P* values less than .008 to be statistically significant (given 6 pairwise comparisons among algorithms). We used Python version 3.6 (Python Software Foundation) to implement the SVM, RNN, and CNN (PyTorch implementation of SqueezeNet) and R version 3.5.1 (R Foundation for Statistical Computing) to evaluate algorithm performance.

## Results

Table 1 shows the number of instances of each phase in our data set. Performance of all 5 algorithms differed based on the global measure used to evaluate them. Accuracy, which is commonly reported in machine learning literature as a primary measure of algorithm performance, was greater than 0.910 for all 5 algorithms on average across phases. Unweighted accuracy ranged between 0.915 and 0.959 across phases and between 0.841 and 0.989 within phases (Table 2 and Table 3).

**Table 1.** Instances of Each Phase Within Cataract Surgery in Data Set

Phase in Cataract Surgery	Faculty Surgeon, No. (%)	Trainee Surgeon, No. (%)	Total, No.
Incision			
Side	31 (26.5)	86 (73.5)	117
Main	40 (30.5)	91 (69.5)	131
Capsulorrhexis	29 (28.7)	72 (71.3)	101
Hydrodissection	28 (28.5)	70 (71.5)	98
Phacoemulsification	31 (29.8)	73 (70.2)	104
Cortical removal	35 (28.9)	86 (71.1)	121
Lens insertion	31 (28.7)	77 (71.3)	108
Ocular viscoelastic device removal	33 (30.0)	77 (70.0)	110
Wound closure			
Corneal hydration	35 (27.3)	93 (72.7)	128
Suture incision	17 (29.3)	41 (70.7)	58

**Table 2.** Summary Measures of Algorithm Performance for Phase Classification

Metric	SVM, Algorithm 1, Instrument Labels	RNN, Algorithm 2, Instrument Labels	CNN, Algorithm 3, Images	CNN-RNN, Algorithm 4, Images	CNN-RNN, Algorithm 5, Images and Instrument Labels
Unweighted accuracy (95% CI)	0.938 (0.937-0.939)	0.959 (0.958-0.960)	0.956 (0.954-0.957)	0.921 (0.920-0.923)	0.915 (0.913-0.916)
Frequency-weighted accuracy (95% CI)	0.935 (0.934-0.936)	0.957 (0.956-0.958)	0.955 (0.953-0.956)	0.919 (0.918-0.920)	0.913 (0.912-0.914)
Inverse variance−weighted accuracy (95% CI)	0.963 (0.962-0.965)	0.976 (0.975-0.978)	0.958 (0.957-0.960)	0.928 (0.926-0.930)	0.920 (0.918-0.922)
Unweighted AUC (95% CI)	0.737 (0.730-0.744)	0.773 (0.770-0.776)	0.712 (0.704-0.719)	0.752 (0.750-0.755)	0.737 (0.735-0.739)

Abbreviations: AUC, area under the receiver operating characteristic curve; CNN, convolutional neural network; RNN, recurrent neural network; SVM, support vector machine.

**Table 3.** Accuracy, Sensitivity, Specificity, and Precision for Algorithms Across Phases

Algorithm and Measure	Side Incision	Main Incision	Capsulorrhexis	Hydrodissection	Phacoemulsification	Cortical Removal	Lens Insertion	Ocular Viscoelastic Device Removal	Wound Closure, Corneal Hydration	Wound Closure, Suture Incision
SVM, algorithm 1, instrument labels (95% CI)										
Accuracy	0.985 (0.982-0.987)	0.930 (0.927-0.933)	0.963 (0.962-0.964)	0.910 (0.907-0.914)	0.958 (0.955-0.961)	0.882 (0.878-0.886)	0.987 (0.986-0.988)	0.899 (0.897-0.900)	0.893 (0.892-0.895)	0.968 (0.964-0.971)
Sensitivity	0.936 (0.917-0.955)	0.949 (0.949-0.949)	0.890 (0.890-0.890)	0.809 (0.799-0.819)	0.904 (0.894-0.914)	0.475 (0.446-0.504)	0.920 (0.920-0.920)	0.247 (0.247-0.247)	0.005 (0.000-0.015)	0.852 (0.784-0.920)
Specificity	0.990 (0.989-0.991)	0.927 (0.924-0.931)	0.972 (0.971-0.973)	0.923 (0.919-0.926)	0.965 (0.962-0.968)	0.932 (0.930-0.934)	0.996 (0.994-0.997)	0.976 (0.975-0.978)	0.999 (0.998-1.000)	0.972 (0.970-0.975)
Precision	0.906 (0.896-0.916)	0.615 (0.604-0.625)	0.798 (0.791-0.805)	0.555 (0.542-0.568)	0.759 (0.742-0.776)	0.459 (0.442-0.476)	0.963 (0.953-0.973)	0.556 (0.539-0.573)	0.517 (0.000-1.000)	0.553 (0.524-0.582)
RNN, algorithm 2, instrument labels (95% CI)										
Accuracy	0.989 (0.987-0.991)	0.985 (0.982-0.987)	0.973 (0.971-0.976)	0.960 (0.957-0.962)	0.962 (0.959-0.965)	0.915 (0.911-0.919)	0.988 (0.987-0.990)	0.927 (0.924-0.930)	0.909 (0.905-0.914)	0.984 (0.981-0.987)
Sensitivity	0.940 (0.925-0.956)	0.974 (0.957-0.991)	0.925 (0.915-0.935)	0.716 (0.706-0.727)	0.812 (0.795-0.829)	0.583 (0.552-0.614)	0.943 (0.934-0.952)	0.508 (0.494-0.521)	0.765 (0.745-0.784)	0.800 (0.728-0.873)
Specificity	0.994 (0.992-0.996)	0.986 (0.985-0.987)	0.979 (0.977-0.981)	0.989 (0.986-0.991)	0.980 (0.978-0.983)	0.956 (0.952-0.959)	0.994 (0.992-0.996)	0.977 (0.974-0.980)	0.926 (0.922-0.931)	0.991 (0.990-0.993)
Precision	0.942 (0.927-0.957)	0.893 (0.885-0.902)	0.847 (0.834-0.860)	0.883 (0.859-0.907)	0.836 (0.819-0.852)	0.615 (0.594-0.637)	0.951 (0.938-0.964)	0.722 (0.695-0.749)	0.554 (0.537-0.570)	0.781 (0.749-0.813)
CNN, algorithm 3, images (95% CI)										
Accuracy	0.962 (0.958-0.966)	0.970 (0.966-0.974)	0.928 (0.923-0.932)	0.957 (0.955-0.958)	0.959 (0.956-0.962)	0.940 (0.935-0.944)	0.964 (0.960-0.967)	0.959 (0.956-0.963)	0.953 (0.948-0.958)	0.966 (0.963-0.970)
Sensitivity	0.723 (0.689-0.756)	0.870 (0.845-0.895)	0.920 (0.920-0.920)	0.623 (0.613-0.634)	0.884 (0.874-0.894)	0.793 (0.762-0.823)	0.813 (0.796-0.830)	0.799 (0.782-0.816)	0.749 (0.722-0.775)	0.279 (0.210-0.347)
Specificity	0.987 (0.984-0.990)	0.982 (0.979-0.985)	0.929 (0.924-0.934)	0.996 (0.995-0.998)	0.968 (0.965-0.971)	0.958 (0.954-0.961)	0.982 (0.979-0.985)	0.978 (0.975-0.982)	0.978 (0.973-0.982)	0.994 (0.992-0.996)
Precision	0.858 (0.829-0.886)	0.856 (0.835-0.877)	0.614 (0.597-0.630)	0.952 (0.932-0.972)	0.770 (0.753-0.787)	0.696 (0.677-0.716)	0.850 (0.828-0.873)	0.816 (0.793-0.838)	0.802 (0.769-0.834)	0.646 (0.555-0.738)
CNN-RNN, algorithm 4, images (95% CI)										
Accuracy	0.939 (0.934-0.945)	0.930 (0.926-0.935)	0.931 (0.928-0.934)	0.936 (0.932-0.939)	0.938 (0.935-0.940)	0.841 (0.837-0.845)	0.930 (0.927-0.933)	0.900 (0.896-0.903)	0.916 (0.911-0.921)	0.951 (0.948-0.955)
Sensitivity	0.609 (0.569-0.649)	0.745 (0.720-0.770)	0.745 (0.735-0.755)	0.557 (0.557-0.557)	0.692 (0.682-0.702)	0.546 (0.521-0.571)	0.486 (0.477-0.496)	0.545 (0.531-0.558)	0.594 (0.568-0.621)	0.414 (0.352-0.477)
Specificity	0.974 (0.971-0.978)	0.953 (0.949-0.957)	0.954 (0.950-0.957)	0.981 (0.977-0.984)	0.968 (0.965-0.970)	0.877 (0.874-0.880)	0.984 (0.982-0.987)	0.942 (0.939-0.946)	0.955 (0.95-0.959)	0.973 (0.970-0.976)
Precision	0.715 (0.684-0.747)	0.659 (0.639-0.679)	0.665 (0.649-0.682)	0.775 (0.743-0.806)	0.723 (0.709-0.737)	0.351 (0.340-0.363)	0.795 (0.764-0.825)	0.529 (0.513-0.545)	0.613 (0.586-0.640)	0.378 (0.334-0.422)
CNN-RNN, algorithm 5, images and instrument labels (95% CI)										
Accuracy	0.932 (0.927-0.937)	0.947 (0.943-0.951)	0.902 (0.899-0.906)	0.900 (0.896-0.904)	0.920 (0.918-0.922)	0.861 (0.858-0.865)	0.914 (0.910-0.917)	0.909 (0.905-0.913)	0.925 (0.922-0.929)	0.937 (0.933-0.941)
Sensitivity	0.512 (0.475-0.550)	0.709 (0.681-0.737)	0.625 (0.615-0.635)	0.495 (0.495-0.495)	0.666 (0.653-0.680)	0.520 (0.503-0.537)	0.547 (0.530-0.563)	0.619 (0.597-0.642)	0.518 (0.499-0.537)	0.421 (0.338-0.503)
Specificity	0.976 (0.973-0.980)	0.976 (0.973-0.979)	0.937 (0.933-0.940)	0.949 (0.944-0.953)	0.951 (0.949-0.953)	0.903 (0.900-0.906)	0.959 (0.956-0.963)	0.943 (0.940-0.947)	0.974 (0.970-0.978)	0.957 (0.955-0.960)
Precision	0.697 (0.661-0.734)	0.782 (0.762-0.802)	0.549 (0.535-0.564)	0.535 (0.513-0.558)	0.624 (0.615-0.633)	0.396 (0.385-0.407)	0.624 (0.603-0.644)	0.566 (0.549-0.583)	0.704 (0.674-0.733)	0.283 (0.241-0.325)

Abbreviations: CNN, convolutional neural network; RNN, recurrent neural network; SVM, support vector machine.

**Figure 2.**
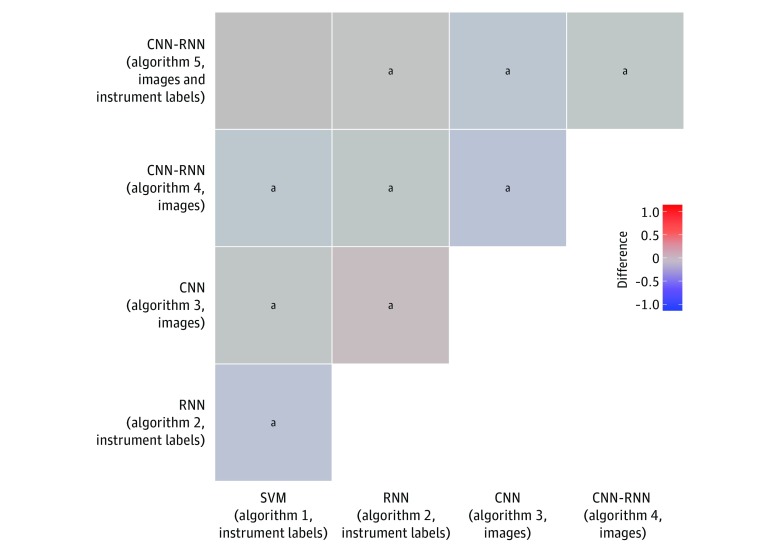
Differences in Area Under the Receiver Operating Characteristic Curve Between Pairs of Algorithms for Phase Classification Area under the receiver operating characteristic curve of algorithm in column subtracted from area under the receiver operating characteristic curve of algorithm in row. CNN indicates convolutional neural network; RNN, recurrent neural network; and SVM, support vector machine. ^a^*P* = .008.

On the other hand, the AUC was within a narrow range (between 0.712 and 0.773) across the 5 algorithms. In pairwise comparisons of algorithms, the magnitude of differences in AUCs was very small, although some of the differences were statistically significant (Figure 2; eTable 2 in the Supplement). Specifically, compared with modeling cross-sectional snapshots of instrument labels (algorithm 1), temporal modeling instrument labels as time-series data (algorithm 2) improved AUC (0.737 vs 0.773; difference, 0.036; 95% CI, 0.044 to 0.029). Algorithm 2 was also associated with a higher AUC compared with modeling spatial features in images with a CNN (algorithm 3; 0.773 vs 0.712; difference, 0.062; 95% CI, 0.054 to 0.069), spatiotemporal models of images with a CNN-RNN pipeline (algorithm 4; 0.773 vs 0.752; difference, 0.021; 95% CI, 0.017 to 0.025), and spatiotemporal models of images plus instrument labels (algorithm 5; 0.773 vs 0.737; difference, 0.037; 95% CI, 0.033 to 0.040). Furthermore, the AUC was lower when modeling spatial features in images with a CNN (algorithm 3) compared with spatiotemporal modeling images with a CNN-RNN pipeline (algorithm 4; 0.712 vs 0.752; difference −0.040; 95% CI, −0.049 to −0.033) and spatiotemporal models of images plus instrument labels (algorithm 5; 0.712 vs 0.737; difference, −0.025; 95% CI, −0.033 to −0.017). Spatiotemporal modeling images with a CNN-RNN (algorithm 4) yielded a higher AUC compared with spatiotemporal models of images plus instrument labels (algorithm 5; 0.752 vs 0.737; difference, 0.016; 95% CI, 0.014 to 0.018).

Phase-level measures, such as sensitivity, further support the notion that performance of the 5 algorithms varied across phases. Sensitivity ranged between 0.005 (95% CI, 0.000 to 0.015) for the SVM for wound closure (corneal hydration) and 0.974 (95% CI, 0.957 to 0.991) for the RNN for main incision. For some phases, such as the side incision and main incision, sensitivity was higher with models of instrument labels (algorithms 1 and 2) than with models of image features with or without instrument labels (algorithms 3, 4, and 5). On the other hand, for phases such as capsulorrhexis, cortical removal, lens insertion, and ophthalmic viscosurgical device removal, modeling spatial features in images (algorithm 3) yielded higher sensitivity than temporal modeling of the spatial features (algorithms 4 and 5). For other phases, such as lens insertion, algorithms for temporal modeling of image features (algorithms 4 and 5) yielded lower sensitivity than those that modeled spatial features in instrument labels or images (algorithms 1 and 3). Furthermore, algorithms relying on video images (algorithms 3, 4, and 5) had lower sensitivity for identifying the wound closure (suture incision) phase. Unlike sensitivity, we observed specificity to be uniformly greater than 0.875 (range, 0.877-0.999) for all phases across all 5 algorithms. Finally, precision was heterogeneous across phases for all 5 algorithms (range, 0.283-0.963), but it was consistently low for cortical removal for all algorithms (Table 3).

## Discussion

Our findings suggest that it is more useful to model instrument labels (alone or with video images) (algorithms 1, 2, 4, and 5) for the automated identification of phases in cataract surgery than video images alone (algorithm 3) (Figure 1). Furthermore, temporal modeling of instrument labels (algorithm 2) appeared to perform better than analyzing cross-sectional snapshots of instruments (algorithm 1). These findings are intuitive because nearly all phases in cataract surgery are performed with instruments that are unique to the phase. For example, a cystatome and/or Utrata forceps are used in capsulorrhexis but not in other phases. Thus, it should be feasible for an expert surgeon or a trained annotator to recognize the phase in any given video by observing the instruments in use. However, some phases share instruments, and either spatial or temporal information can be discriminative in such situations. For example, the irrigation aspiration cannula is used for cortical removal as well as ophthalmic viscosurgical device removal. In this case, contextual information, such as the presence of an artificial lens, can be useful to disambiguate between the 2 phases.

Our work is a consolidated study of current deep learning techniques for phase classification in cataract surgery. To our knowledge, only 1 other study^16^ used modeling videos of cataract surgery presegmented into phases with CNNs. Using a data set of 21 videos, Primus et al^16^ trained a GoogLeNet to assign frames to phases. GoogLeNet (Google) is based on a different neural network architecture than SqueezeNet, but both are pretrained on the same data set (ImageNet; Stanford Vision Lab, Stanford University; and Princeton University). Unlike our study, Primus et al^16^ only modeled images and reported sensitivity estimates that were heterogeneous across different phases. Another study^14^ used deep learning techniques to simultaneously segment phase boundaries and assign phase labels for cataract surgery, reporting 78.28% frame-level accuracy. However, their approach differed from ours by first identifying the instrument with a CNN, then applying CNN features to segment phases and label them. Other studies used various machine learning techniques, such as an SVM applied to abstract features describing images (eg, image texture),^11,12,13^ spatiotemporal polynomials applied to optical flow across the video,^20^ content-based video retrieval using hidden Markov models,^10,21^ conditional random fields,^10^ or Bayesian networks^10^ applied to features, such as motion histograms computed within subsequences of the video.^13^ Findings from these other studies are consistent with those we reported in this study in terms of heterogeneity in performance metrics across phases.

Our findings are significant in the context of both crowdsourced and automated detection of instruments in cataract surgery videos. In 1 study,^22^ a crowd of raters recruited from the Amazon Mechanical Turk platform identified a select subset of instruments used in cataract surgery with an accuracy of 0.89 (against expert labels) and moderate interannotator reliability (Fleiss κ of 0.63). Separately, Bouget et al^15^ applied conventional computer vision techniques to segment and identify instruments in video images from cataract surgery. More recently, Charrière et al^10^ reported an AUC of 0.986 when using statistical techniques such as hidden Markov models, conditional random fields, and Bayesian networks to detect the presence or absence of an instrument in cataract surgery videos. In addition, Prellberg and Kramer^23^ applied CNNs to identify instruments using video images of cataract surgery and reported an estimated AUC of 0.997. Similarly, Al Hajj et al^24^ applied a CNN-RNN analysis pipeline to identify instruments in cataract surgery videos and reported an estimated AUC of 0.996. In summary, it is now possible to efficiently obtain accurate annotations on surgical instruments in videos of cataract surgery that can be used in different applications for surgical training.

One question our work poses is how best to specify ground truth to evaluate algorithms for phases in cataract surgery to be useful for education. While it may eventually be possible for an algorithm to always correctly detect phases in cataract surgery, we postulate that the algorithm should at least match human-level performance. The conventional approach to specifying human-level performance for activity-recognition algorithms is to describe measures of reliability among multiple annotators who typically mark the start and end of each phase in a video of a procedure. In other words, the annotators perform a combined segmentation and classification. In cataract surgery and other procedures where instrument labels have distinct information on phases, we believe that multiple approaches are necessary to establish human-level performance. These approaches correspond to the experiments described in this work: human annotation of the phase given a snapshot of instruments in use, time series of instrument labels, and images. Annotations may be obtained either from expert surgeons, which is not always feasible, or from untrained individuals via crowdsourcing. It may be possible to process video images that omit the instrument to generate annotations with spatiotemporal information in videos with and without instruments, but it is nontrivial to implement at scale.

Our work also emphasizes prespecification of adequate algorithm performance. To be useful for surgical education, further advances in technology for automated phase detection should be guided by the needs of surgeons, surgical educators, and trainees. An important application of automated phase detection is to provide objective, phase-specific assessments of surgical technical skill. For this purpose, algorithms should be able to take videos of the surgical field (either in real time or offline) as input, find boundaries between phases, and assign each segment a phase label. There is considerable room for improvement in these algorithms based on findings from our study and related studies. From a methodological perspective, it is unclear how imperfect algorithms for phase segmentation and classification could affect downstream applications.

While it may be possible to train phase-detecting algorithms to near-perfect accuracy in a given data set, it is essential to evaluate them across data sets. In this context, data science for eye care can progress with shared data sets, such as Cataract 101.^25^ However, shared data sets become useful when they are uniform in critical aspects (eg, annotations) and when they capture heterogeneity in data across clinical settings (eg, variation in instruments across hospitals and their use in different phases across surgeons). Finally, adequacy of algorithm performance should be determined in terms of its utility for and effect on downstream applications, such as objective technical skill assessment.

### Limitations

Our study has limitations. We evaluated algorithms’ ability to assign labels to a presegmented video, but real-life applications will require algorithms to both detect segment boundaries and assign phase labels. Our work represents an initial step toward a comprehensive phase-recognition algorithm for cataract surgery. In our analyses, we did not account for the fact that multiple videos in our data set captured procedures performed by the same surgeons. However, it is unlikely that our estimates are biased because we anticipate that the performance of algorithms for phase classification, unlike that of algorithms for skill assessment, is not influenced by surgeon-specific style. We did not annotate all instruments used in the procedure, which may have affected the performance of some of the algorithms. For example, we did not label the anterior chamber cannula or the instrument used for corneal hydration during wound closure. For this reason, the input to the SVM (algorithm 1) for this phase identified no instrument in use. This aberration in input explains the low sensitivity observed with the SVM when identifying the wound closure (corneal hydration) phase. Using accuracy, time-series modeling of tool labels alone (algorithm 2) appeared to yield adequate information to identify most phases compared with modeling images with a CNN (algorithm 3). However, the AUC across all 5 analyses ranged between 0.712 and 0.773, suggesting there is considerable room for improvement. Also, we used a convenience sample of videos of cataract surgery with multiple records per surgeon. Validation with an independent data set containing videos of procedures by different surgeons and in different clinical contexts is necessary to verify the classification error observed in this study. In a broader context, while instruments in use reveal much about the phases of cataract surgery, this may not hold true for other surgical procedures.

## Conclusions

In cataract procedures, instrument labels are informative for machine learning and deep learning algorithms to identify different phases. Deep learning algorithms for spatiotemporal modeling of video images may be more accurate for identifying presegmented phases than modeling spatial aspects of video images.

## Notes

Supplement. **eAppendix.** Supplemental methods **eTable 1.** Metrics to evaluate performance of algorithms to classify phases in cataract surgery **eTable 2.** Differences in area under the receiver operating characteristic curve between pairs of algorithms for phase classification
