# Supplementary material for: Assessment of Automated Identification of Phases in Videos of Cataract Surgery Using Machine Learning and Deep Learning Techniques
Source: JAMA Netw Open. 2019 Apr 5;2(4):e191860. doi: 10.1001/jamanetworkopen.2019.1860 (PMC6450320; doi:10.1001/jamanetworkopen.2019.1860)
Supplement: Supplement. — eAppendix. Supplemental methods eTable 1. Metrics to evaluate performance of algorithms to classify phases in cataract surgery eTable 2. Differences in area under the receiver operating characteristic curve between pairs of algorithms for phase classification [file jamanetwopen-2-e191860-s001.pdf]

Supplementary Online Content

Yu F, Silva Croso G, Kim TS, et al. Assessment of automated identification of phases in videos of cataract surgery using machine learning and deep learning techniques. *JAMA Netw Open.* 2019;2(4):e191860. doi:10.1001/jamanetworkopen.2019.1860

eAppendix. Supplemental methods
eTable 1. Metrics to evaluate performance of algorithms to classify phases in cataract surgery
eTable 2. Differences in area under the receiver operating characteristic curve between pairs of algorithms for phase classification

This supplementary material has been provided by the authors to give readers additional information about their work.

© 2019 Yu F et al. *JAMA Network Open.*

eAppendix. Supplemental methods

This section supplements the Methods described in the manuscript to provide sufficient detail to replicate our experiments. We used Python 3.5.2 with OpenCV and skvideo.io packages for video processing, scikit-learn for nearest neighbors, and PyTorch 0.4.1 as our deep learning Python library.

Support Vector Machine (SVM)

We used a SVM in Algorithm #1. The input to the SVM is labels for instruments that are in use at a given image or frame in a video, (i.e., cross-sectional data). We adopted a one-vs-the-rest strategy to implement the SVM, i.e., we fitted one SVM per phase to classify whether a given vector of instrument labels (in use at the time) belongs to the particular phase or any of the other phases. We fitted 10 such classifiers, one for each phase.

We represented labels of instruments in use in a given video frame as a D dimensional indicator vector where the d-th dimension is 1 if the corresponding tool is in use. Let $X_V^P = \{x_{1\ldots n}\}_V^P$ be a set of n labels of instruments in use within phase P of video clip V. We select a subset $X_V^{P*} = \{x_{1\ldots n}^*\}_V^P$ from X_V^P such that X_V^{P*} is a unique set of m features. For example, if a particular phase contained only two unique combinations of instrument label, we included the two as part of the training data. The collection of X_V^{P*} for all Ps and Vs constituted the dataset to fit our multi-class SVMs.

We used a linear SVM implementation provided by sklearn and performed a grid search over the penalty parameters $C \in \{0.1, 0.2, 0.5, 1, 2, 5\}$ of the error terms.

Temporal Recurrent Neural Network (RNN)

We used the RNN in Algorithms #2, #4, and #5. For the RNN, we implemented a standard long short-term memory (LSTM) model. We implemented three separate RNN models with different inputs:

1. Spatial features obtained from the spatial CNN
2. Spatial features obtained from the spatial CNN appending instrument labels
3. Instrument labels alone

Given a single frame in the video, the output dimension of SqueezeNet (spatial CNN model) is a vector of size 512 and the dimension of the instrument labels (annotations) is 14. Thus, the inputs for the three separate RNN models itemized above are 512 by temporal length T, 526 by temporal length T, and 14 by temporal length T, respectively. We structure the RNN as follows:

- An LSTM layer that outputs a 512 dimensional vector. The input at each time-step is fed into this layer and saved.

© 2019 Yu F et al. *JAMA Network Open.*

- A global average pooling across time-steps.
- A fully connected layer that outputs a feature vector of length 128.
- The resulting feature is trained to classify phase of the clip using softmax activation and cross-entropy loss.

The following are the optimization parameters to replicate our study. We optimize the temporal RNN using the ADAM optimizer with the following parameters:

- Learning Rate: 0.001
- Weight Decay: 1e-4
- α: 0.1
- Number of Epochs: 100

Spatial Convolutional neural network (CNN)

We used the CNN in Algorithms #3, #4, and #5. To implement the spatial CNN, we utilized the SqueezeNet architecture. SqueezeNet consists of multiple fire modules. Each module contains a series of $\boldsymbol{s_1}$ 1×1 convolutional filters to "squeeze" the information followed by a set of $\boldsymbol{e_1}$ 1×1 and $\boldsymbol{e_3}$ 3×3 filters to expand. In the standard architecture, there are 8 fire modules. The number of filters in each of the modules is given in Figure 1. SqueezeNet is implemented as follows:

1. A convolutional layer with 96 7×7 filters
2. module 1 through module 3
3. Max pooling
4. module 4 through module 7
5. Max pooling
6. module 8
7. Convolution with n filters, where n is the number of classes. In our case, n is 10.
8. Global Average Pooling
9. Softmax.

© 2019 Yu F et al. *JAMA Network Open.*

	s_1	e_1	e_3
Module 1	16	64	64
Module 2	16	64	64
Module 3	32	128	128
Module 4	32	128	128
Module 5	48	192	192
Module 6	48	192	192
Module 7	64	256	256
Module 8	64	256	256

Figure 1: Information on the number of filters in each fire module. Note that in the SqueezeNet paper, these modules are labeled ”fire2” through ”fire9”.

We take PyTorch’s implementation of SqueezeNet from their pretrainedmodels package, already pre-trained on ImageNet. We then replaced the last convolutional layer with one that has 10 classes rather than 1000 in order to allow for classification of the 10 phases. The model is then finetuned to classify phase on cataract surgery images. To do this, we first extract training examples from our videos through the following steps.

- One frame per second is extracted from all surgical videos. This is done while still maintaining the split between training and evaluation data.
- This image training data is then balanced so there is an even distribution of images for each phase.

We then finetuned the model on the above data set using the ADAM optimizer with the following parameters:

- Initial Learning Rate: 0.001
- Decay: 1e-4
- Momentum: 0.9
- Batch Size: 128
- Number of Epochs: 100

© 2019 Yu F et al. *JAMA Network Open.*

eTables

eTable 1. Metrics to evaluate performance of algorithms to classify phases in cataract surgery

Metric	Interpretation
Sensitivity[1]	True positive ratio; probability of assigning the correct phase label to a frame from a given phase
Precision[1]	Positive predictive value; probability that a frame assigned a certain phase label belongs to that phase
Specificity[1]	True negative ratio; probability of assigning a frame that does not belong to a certain phase with a different phase label
Accuracy – Unweighted average[2]	Simple average of phase-specific accuracies
Accuracy – Weighted average[2]	Average of phase-specific accuracies weighted by frequency of instances of the phase in the data set; and inverse variance weights
Area under the receiver operating characteristic curve[2]	Probability that a randomly chosen instance of a given phase is assigned a label that is more likely to belong to the correct phase than a randomly chosen instance of a different phase
1 – specific to each phase; 2 – global; across phases	

© 2019 Yu F et al. *JAMA Network Open.*

eTable 2. Differences in area under the receiver operating characteristic curve between pairs of algorithms for phase classification (95% confidence intervals in parentheses; difference in AUC with algorithm in column subtracted from AUC with algorithm in row)

	**RNN (instrument labels)**	**CNN (images)**	**CNN-RNN (images)**	**CNN-RNN (images & instrument labels)**
**SVM (instrument labels)**	-0.036 (-0.044 to -0.029); P < 0.0001	0.025 (0.015 to 0.035); P < 0.0001	-0.016 (-0.023 to -0.008); P < 0.0001	0 (-0.007 to 0.007); P = 0.9595
**RNN (instrument labels)**	-	0.062 (0.054 to 0.069); P < 0.0001	0.021 (0.017 to 0.025); P < 0.0001	0.037 (0.033 to 0.04); P < 0.0001
**CNN (images)**	-	-	-0.040 (-0.049 to -0.033); P < 0.0001	-0.025 (-0.033 to -0.017); P < 0.0001
**CNN-RNN (images)**	-	-	-	0.016 (0.014 to 0.018); P < 0.0001

© 2019 Yu F et al. *JAMA Network Open.*
